# Reliability analysis of successive Corvis ST® measurements in keratoconus 2 years after accelerated corneal crosslinking compared to untreated keratoconus corneas

**DOI:** 10.1007/s00417-022-05881-6

**Published:** 2022-10-28

**Authors:** Kassandra Xanthopoulou, Berthold Seitz, Michael W. Belin, Elias Flockerzi

**Affiliations:** 1 Department of Ophthalmology, Saarland University Medical Center, Homburg, Germany; 2 Department of Ophthalmology & Vision Science, University of Arizona, Tucson, AZ USA

**Keywords:** Corneal biomechanics, Corvis, Keratoconus, Corneal crosslinking, Biomechanical E-staging

## Abstract

**Purpose:**

To assess the reliability of successive Corvis ST® measurements (CST, Oculus, Wetzlar, Germany) in keratoconus (KC) ≥ 2 years after accelerated corneal crosslinking (9 mW/cm^2^, 10 min, 5.4 J/cm^2^) compared to untreated KC corneas.

**Methods:**

Three successive CST measurements per eye were performed in ≥ 2 years after CXL (CXLG, *n* = 20 corneas of 16 patients) and a control group consisting of non-operated, ABC-stage-matched KC corneas according to Belin’s ABCD KC grading (controls, *n* = 20 corneas, 20 patients). Main outcome measures included maximal keratometry (Kmax), the Belin/Ambrósio-Enhanced-Ectasia-Deviation-Index BAD-D; the biomechanical parameters A1 velocity, deformation amplitude (DA) ratio 2 mm, Ambrósio relational thickness to the horizontal profile (ARTh), integrated radius, stiffness parameter A1 (SP-A1), and the Corvis Biomechanical Factor (CBiF, the linearized term of the Corvis Biomechanical Index). Mean values, standard deviations, and Cronbach’s alpha (CA) were calculated.

**Results:**

Both groups were tomographically comparable (BAD: 11.5 ± 4.7|11.2 ± 3.6, *p* = 0.682, Kmax: 60.5 ± 7.2|60.7 ± 7.7, *p* = 0.868 for controls|CXLG, paired *t*-test). A1 velocity (mean ± SD: 0.176 ± 0.02|0.183 ± 0.02, *p* = 0.090, CA: 0.960|0.960), DA ratio 2 mm (6.04 ± 1.13|6.14 ± 1.03, *p* = 0.490, CA: 0.967|0.967), integrated radius (12.08 ± 2.5|12.42 ± 1.9, *p* = 0.450, CA: 0.976|0.976), and CBiF (4.62 ± 0.6|4.62 ± 0.4, *p* = 0.830, CA: 0.965|0.965) were also comparable (controls|CXLG). ARTh was significantly higher in controls (177.1 ± 59, CA: 0.993) than after CXL (155.21 ± 65, *p* = 0.0062, CA: 0.993) and SP-A1 was significantly higher after CXL (59.2 ± 13, CA: 0.912) than in controls (52.2 ± 16, *p* = 0.0018, CA: 0.912).

**Conclusion:**

ARTh and SP-A1 differed significantly between controls and CXLG. Biomechanical measurements were generally of excellent reliability in both groups. CXL seems to affect biomechanical measurements of human corneas over more than 2 years.

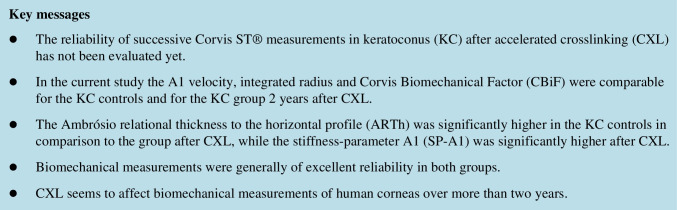

## Introduction

Keratoconus (KC) is an ectatic corneal disease that can be diagnosed based on the abnormal corneal biomechanical response to mechanical stress. Several studies investigated corneal deformation and found KC to show higher deformation amplitudes compared to healthy corneas [1, 2].

The Corneal Visualization Scheimpflug Technology Corvis ST® (CST, Oculus, Wetzlar, Germany) is a non-contact pneumotonometer that analyzes the corneal deformation response to a standardized air puff using an ultra-high-speed Scheimpflug camera that captures over 4300 frames per second [3, 4]. The Corvis Biomechanical Index (CBI) was designed to differentiate between ectatic and normal corneas [5]. It is reported to consist of a combination of dynamic corneal response (DCR) parameters: (1) the speed of the corneal apex at inward applanation A1 (A1 velocity), (2) the maximum value of the ratio between the deformation amplitude at the apex and 2 mm from the center (DA Ratio 2 mm), (3) Ambrósio’s relational thickness through the horizontal profile (ARTh), (4) the radius of curvature during the concave phase of the deformation (integrated radius), and (5) the stiffness parameter at inward applanation A1 (SP-A1) [1, 5]. The Corvis Biomechanical Factor (CBiF) is the linearized term of the CBI [6] and a biomechanical E-staging for KC and ectatic corneal diseases with stages E0 to E4 was developed by dividing the CBiF value range into five groups to augment the existing tomographic ABCD KC grading system [7]. The ABCD KC grading system itself was introduced in 2016 and is based on anterior (“A”) and posterior (“B”) radius of curvature measured over a 3.0 mm zone centered on the thinnest corneal pachymetry (“C”) and it also includes best-spectacle-corrected visual acuity (“D”) [8].

Analysis of corneal biomechanics has shown that corneal cross-linking (CXL) leads to an increase in corneal stiffness and a reduced maximal deformation [9, 10]. The ultraviolet (UV) corneal crosslinking (CXL) with riboflavin instillation results in an increased corneal rigidity and stiffening in a minimally invasive way, thus reducing the steepness and halting progression [11, 12]. Accelerated epithelium-off (epi-off) CXL (9 mW/cm^2^, 10 min, fluence 5.4 J/cm^2^) has been proven to have the same long-term efficacy as the standard CXL procedure (3 mW/cm^2^, 30 min, fluence 5.4 J/cm^2^) [13, 14].

This study aimed to assess the reliability of biomechanical corneal analysis based on three successive CST measurements in two groups of KC patients. Group 1 consisted of patients who underwent epithelium-off accelerated corneal CXL with a minimum 2-year follow-up (crosslinking group, CXLG). Group 2 consisted of non-operated, ABC-stage-matched KC patients serving as controls (controls).

## Patients and methods

This retrospective cross-sectional cohort study was performed at the Department of Ophthalmology at Saarland University Medical Center in Homburg, Germany. The patient charts of KC patients ≥ 18 years were enrolled from the Homburg Keratoconus Center (HKC) which was established in 2010 [15]. All patients in the current study signed an informed consent for the use of their data for analysis and therefore participated in the HKC observational clinical study, which was approved by the local ethics committee (Ethikkommission bei der Ärztekammer des Saarlandes, reference number 121/20, trial number NCT03923101, US National Institutes of Health, ClinicalTrials.gov) and respects the principles of the Declaration of Helsinki.

The crosslinking group (CXLG) consisted of 20 KC corneas of 16 KC patients who underwent epithelium-off accelerated CXL (9 mW/cm^2^, 10 min, 5.4 J/cm^2^) two or more years ago with the Avedro KXL® system (Avedro, Waltham, MA, USA) using riboflavin VibeX Rapid™ 0.1% solution (Avedro, Waltham, MA, USA). Diagnosis of KC was based on clinical features such as corneal hemosiderin deposition known as a Fleischer ring, Vogt striae, corneal thinning on the slit lamp examination, and/or tomographic abnormalities as detected within the Belin/Ambrósio-enhanced ectasia screening display within the Pentacam® HR software (high resolution (HR), Oculus, Wetzlar, Germany) [16, 17]. KC progression was defined as an increase in corneal astigmatism ≥ 1 diopter (D), and/or an increase of maximal keratometry (Kmax) ≥ 1 D and/or a decrease of corneal thickness of 30 µm within 1 year. These patients underwent 3 successive CST measurements at 2 or more years after CXL (crosslinking group, CXLG) during one regular follow-up examination.

A control group for biomechanical measurements was formed consisting of 20 non-operated KC corneas of 20 KC patients (controls) that underwent the same examinations. Those corneas were chosen as ABC-stage-matched controls according to Belin’s tomographic ABCD KC grading. Both groups had to stop wearing contact lenses at least 3 days prior to the measurements. First, the ABC parameters were collected for the CXLG from the “topometric/KC staging” display of the Pentacam software. Second, ABC-stage-matched controls were enrolled from the HKC. Consequently, each cornea treated with CXL was paired with an untreated cornea of the same ABC stage as a control.

The ABC stage was derived from Pentacam measurements, which always proceeded the CST measurements to avoid tomographical changes caused by the CST air puff indentation. Both the Pentacam and CST measurements were only included with an “OK” score (in advanced stages, “model deviations” were also accepted) and the measurements were independently reviewed by two physicians (KX, EF).

The maximal keratometry (Kmax) and the Belin/Ambrósio-Enhanced-Ectasia-Deviation-Index BAD-D were analyzed to determine tomographic KC severity in addition to the ABC severity stage. The main outcome DCR parameters included A1 velocity, deformation amplitude (DA) ratio 2 mm, Ambrósio relational thickness to the horizontal profile (ARTh), integrated radius, stiffness parameter A1 (SP-A1), and the Corvis Biomechanical Factor (CBiF, the linearized term of the Corvis Biomechanical Index, CBI). The outcome measures were first analyzed for normal distribution using the Shapiro–Wilk test and assuming a normal distribution with *p* ≥ 0.05. The parameters resulting from three measurements per eye per patient were subsequently compared between the control group and the CXLG using the two-tailed paired *t*-test (if normally distributed) or the Wilcoxon matched-pairs test (if not normally distributed) assuming significant differences with *p* < 0.05. The paired tests were used to obtain the most accurate comparison between the respective crosslinked and stage-matched non-treated control corneas.

The coefficients of repeatability were calculated as the within-subject standard deviation Sw × √2 × 1.96. The intraclass correlation coefficients (ICC) that correlate successive measurements carried out on the same subject or a collective of patients with the same underlying disease and Cronbach’s alpha (CA) were calculated to determine the reliability of the biomechanical measurements.

## Results

The CXLG consisted of 12 right eyes and 8 left eyes. Accelerated CXL was performed on average 48 ± 19 months ago in these corneas. The mean age of the patients in the CXLG was 31 ± 11 years. Eleven right eyes and 9 left eyes were chosen ABC-stage-matched (Table 1) as controls and the mean age of the control patients was 39 ± 14 years. The age of the patients was normally distributed (controls: *p* = 0.805 and CXLG: *p* = 0.111; Shapiro–Wilk test) and did not differ significantly between the control group and the CXLG (*p* = 0.065, paired *t*-test).

**Table 1:** Comparison of the control group and the crosslinking group (CXLG). ABC-stage-matched non-operated KC controls were paired with KC corneas ≥ 2 years after accelerated corneal crosslinking (9 mW/cm^2^, 10 min, 5.4 J/cm^2^). *Kmax* (diopters), maximal keratometry; *ARC* (mm), anterior radius of curvature; *PRC* (mm), posterior radius of curvature; *TCT* (µm), thinnest corneal thickness. *BAD-D*, Belin/Ambrósio-Enhanced-Ectasia-Deviation-Index. *P*-values calculated by paired *t*-test^T^, if normally distributed

	Controls	CXLG	*P*
Gender	13 male, 7 female	13 male, 3 female	
Age	39 ± 14	31 ± 11	0.065^ T^
Kmax	60.5 ± 7	60.7 ± 8	0.868^ T^
ARC	6.2 ± 0.6	6.3 ± 0.6	0.344^ T^
PRC	4.6 ± 0.6	4.5 ± 0.4	0.605^ T^
TCT	456 ± 34	452 ± 27	0.401^ T^
BAD-D	11.5 ± 5	11.3 ± 4	0.682^ T^

Both the controls and CXLG group were tomographically comparable: mean Kmax amounted to 60.5 ± 7.2|60.7 ± 7.7 (controls|CXLG, *p* = 0.868) and mean BAD-D to 11.5 ± 4.7|11.2 ± 3.6 (controls|CXLG, *p* = 0.682, paired *t*-test). The anterior and posterior radii of curvature (ARC, PRC, Table 1) and thinnest corneal thickness (TCT, Table 1) were also without statistical differences and, thus, comparable in both groups.

**Fig. 1:**
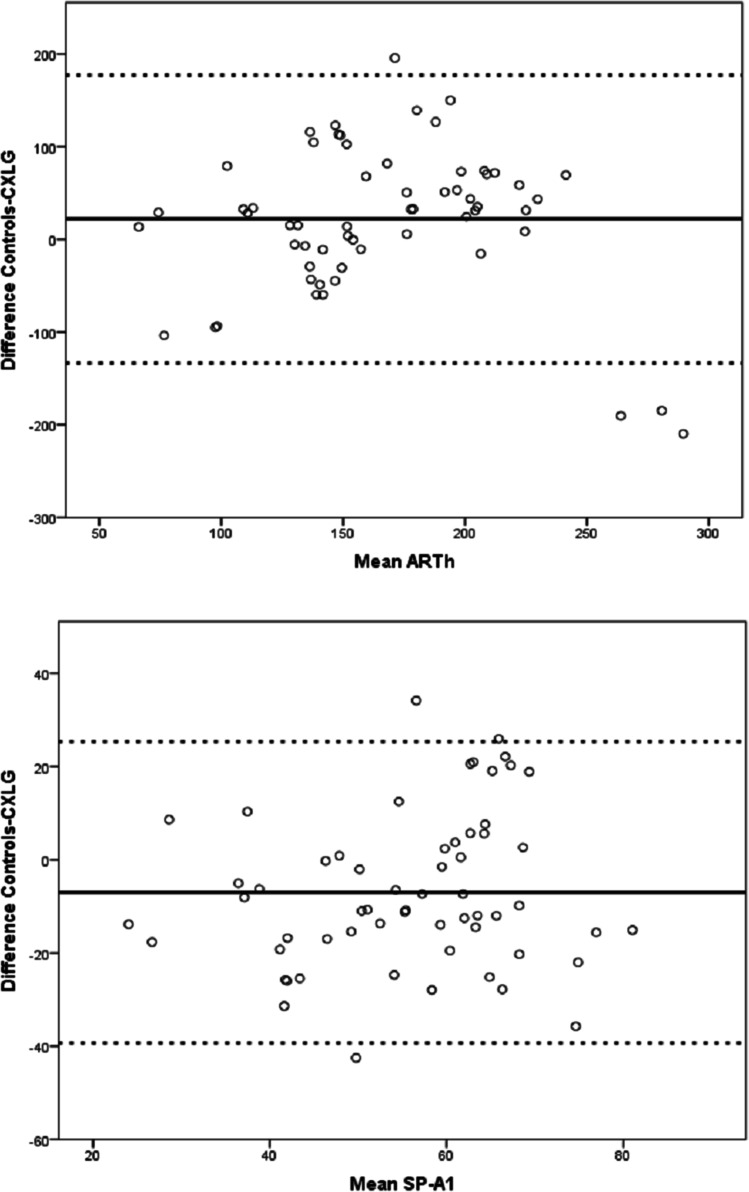
Bland–Altman plots showing the mean difference (solid black line) between controls and CXLG for ARTh and SP-A1 and the 95% limits of agreement (dotted lines)

The mean values of three measurements for A1 velocity, DA ratio 2 mm, integrated radius, and CBiF were also comparable between controls and the CXLG. Mean ARTh was significantly higher in controls (177.1 ± 59) than after CXL (155.21 ± 65, *p* = 0.0062) and mean SP-A1 was significantly higher after CXL (59.2 ± 13) than in controls (52.2 ± 16, *p* = 0.0018, Table 2). Bland–Altman plots were created for the two parameters that differed significantly between controls and the CXLG (ARTh and SP-A1, Fig. 1) showing the mean difference and the 95% limits of agreement.

**Table 2:** Main outcome measures in the control group (controls) and ≥ 2 years after crosslinking group (CXLG). *Mean* ± *SD*, standard deviation resulting out of three measurements per eye; *ICC*, intraclass correlation coefficient; *CA*, Cronbach’s alpha; *DA ratio 2 mm*, deformation amplitude (DA) ratio 2 mm; *ARTh*, Ambrósio relational thickness to the horizontal profile; *SP-A1*, stiffness parameter A1; *CBiF*, Corvis Biomechanical Factor (the linearized term of the Corvis Biomechanical Index, CBI). Comparable values for mean ± SD in controls and CXLG except for ARTh and SP-A1, *p*-values calculated by (1) paired two-tailed *t*-test^T^, if normally distributed or by (2) Wilcoxon matched-pairs test^W^ if not normally distributed—as determined by Shapiro–Wilk test. Coefficients of repeatability for each parameter in controls and CXLG calculated as the within-subject standard deviation Sw × √2 × 1.96. Identical intraclass correlation coefficients and Cronbach’s alpha values in the CXLG and in untreated controls. CA ≥ 0.912 indicating excellent reliability

Parameter	Controls Mean ± SD	CXLG Mean ± SD	P	Controls Coefficient of repeatability	CXLG Coefficient of repeatability	Controls and CXLG ICC	Controls and CXLG CA
A1 velocity	0.176 ± 0.02	0.183 ± 0.02	0.090^ T^	0.01	0.02	0.956	0.960
DA ratio 2 mm	6.04 ± 1.13	6.14 ± 1.03	0.490^ W^	0.97	0.82	0.952	0.967
Integrated radius	12.08 ± 2.5	12.42 ± 1.90	0.450^ W^	2.07	1.28	0.969	0.976
ARTh	177.1 ± 59	155.21 ± 65	0.0062^ W^	33.12	25.00	0.991	0.993
SP-A1	52.2 ± 16	59.2 ± 13	0.0018 T	12.31	16.46	0.894	0.912
CBiF	4.62 ± 0.6	4.62 ± 0.4	0.830^ W^	0.27	0.39	0.955	0.965

The coefficients of repeatability were lower in controls than the CXLG for A1 velocity, SP-A1, and the CBiF and lower in the CXLG than in controls for DA ratio 2 mm, integrated radius, and ARTh (Table 2).

The intraclass correlation coefficients and Cronbach’s alpha values were identical in both the CXLG and controls and indicated an excellent reliability of the biomechanical measurements (CA ≥ 0.912, Table 2).

## Discussion

This study analyzed the reliability of biomechanical CST measurements in KC corneas ≥ 2 years after accelerated corneal cross-linking compared to untreated KC controls of the same ABC stage. Besides other studies, the recent introduction of a biomechanical E-staging for ectatic corneal diseases based on the CBiF [7, 15] raised the question of whether CXL has a detectable long-term effect on corneal biomechanics and on the reliability of biomechanical measurements of the human cornea.

The CST assesses the biomechanical properties of the cornea [1] and its measurements depend on corneal rigidity. An in vitro study reported that corneal indentation and inward versus outward deformation after air puff indentation were reduced significantly after CXL, which indicates that CXL changes the viscoelastic corneal properties and leads to corneal stiffening [18]. In contrast, an in vivo study found no significant differences between pre- and 4 years postoperative biomechanical parameters obtained with the CST system after CXL with exception of the integrated radius [19]. The current study found no significant differences between controls and the CXLG at 2 years postoperatively for the majority of the biomechanical CST parameters that were analyzed: A1 velocity, the speed of the corneal apex at inward applanation, the ratio between the deformation amplitude at the apex and 2 mm away from the center (DA ratio 2 mm), the radius of curvature during the concave phase of the deformation (integrated radius), and CBiF, which is the recently introduced linearized term of the CBI and serves as a basis for the Homburg biomechanical E-staging [7]. All the patients of this study presented during regular follow-up examinations and their tomographic values remained stable after CXL without requiring repeated CXL in the CXLG. Consequently, comparable results of the CBiF between controls and CXLG also indicate a stabilization of the corneas in the CXLG at the level of non-progressive KC corneas within the control group.

Strikingly, there were highly significant differences between controls and CXLG for the biomechanical parameters (1) Ambrósio’s relational thickness through the horizontal profile (ARTh) and (2) the stiffness parameter at inward apö8planation A1 (SP-A1). In contrast, the aforementioned study found no significant differences when comparing those parameters pre- and postoperatively four years after CXL [19].

ARTh results out of the division of the thinnest corneal thickness through the pachymetric progression index and lower values indicate a centrally thinner cornea with a fast thickness increase towards the periphery [5]. This study found significantly lower ARTh values in the CXLG (Table 2), although the controls were selected ABC-stage-matched and thus were comparable with respect to the thinnest corneal thickness. It is known that the thinnest corneal thickness may decrease after CXL [20] which might result in lower ARTh values postoperatively. The lower ARTh value in our CXLG could be due to a coinciding effect of a postoperative decrease in corneal thickness and flattening of the corneal apex as a CXL result [20].

SP-A1 is a parameter that has been reported to increase markedly after CXL as a result of the increasing corneal rigidity [21]. Comparing controls and CXLG, the current study established the largest difference between both groups for SP-A1 with significantly higher values after CXL (Table 2). This could be interpreted as a surrogate measure for CXL efficiency and this indicates that despite (1) the small sample size of this study and (2) a tomographic ABC-matching, biomechanical differences remain measurable even more than 2 years (on average 48 ± 19 months) after CXL.

Although not reaching statistical significance, the result of postoperatively higher SP-A1 values in the long term has also been found by Sedaghat et al. [19] in a long-term follow-up of 18 eyes of 18 KC patients at 4 years after standard CXL according to the Dresden protocol. Interestingly—and in contrast to our results—they found slightly, yet not significantly higher ARTh values 4 years after CXL than preoperatively, which would indicate a centrally thicker cornea with a lower thickness increase towards the periphery. It has still to be determined (1) to what extent and (2) how long postoperatively thickness-related tomographic and biomechanical Scheimpflug-measured parameters are prone to measurement artifacts, and, thus, may lead to seemingly contradictory results.

Several studies confirmed a good to excellent reliability of biomechanical CST measurements in normal and KC eyes [22–24]. Yang et al. [24] found high-reliability values for A1 velocity and DA ratio 2 mm after three sequential measurements in 77 healthy corneas and 77 mild to moderate KC eyes. A recent study evaluated the reliability of CST parameters in untreated KC based on 5 successive CST measurements and found good to excellent reliability independent of the KC stage [25]. The current study examined the reliability of the biomechanical parameter measurements based on three successive CST measurements and (1) the calculation of the intraclass correlation coefficients and (2) the Cronbach’s alpha. The ICC (≥ 0.894) and CA values were identical in both the controls and the CXLG. The CA values ranged from 0.912 (SP-A1) to 0.993 (ARTh) which indicates an excellent reliability [26] of the biomechanical measurements not only in the stabilized CXLG, but also in the untreated controls. Although it was not developed to measure the effect of CXL, the excellent reliability of the CBiF (CA: 0.965 in both controls and CXLG) indicates that this parameter can also be used after CXL to assess KC severity, and therefore biomechanical stability.

Limitations of this study are the small sample size with the majority being moderate to advanced KC stages (Table 1) and the choice of a control group. Ideally, patients would have been followed up with three successive measurements prior to CXL and with 3 successive measurements more than 2 years after CXL. Since these measurements were not available, an ABC-stage-matched control group was created and these controls were tomographically comparable to the CXLG (Table 1) at the time of comparison—which does not take into account differences in duration of KC disease. The primary aim of this study was to analyze the reliability of biomechanical CST measurements in KC corneas ≥ 2 years after accelerated CXL compared to untreated KC corneas—which is why the CXLG consisted of initially progressive KC corneas and the control group of stable KC corneas. The KC progression within the CXLG should have been halted by the CXL effect which should, in turn, mitigate the aspect of different progression rates in both groups. Another limitation is the inclusion of two eyes per patient in eight cases in the CXLG. This could lead to bias, as two eyes of one patient are not considered to be independent.

In summary, we found an excellent reliability of the biomechanical measurements in KC corneas ≥ 2 years after CXL and in untreated KC corneas of the same ABC stage. Significant differences between both groups were found for (1) ARTh (controls > CXLG, *p* = 0.0062, Table 2) and (2) SP-A1 (CXLG > controls, *p* = 0.0018, Table 2). Together with a CXL-induced stiffening effect, this may be attributable to a postoperative decrease in corneal thickness and flattening of the corneal apex. This study thus indicates that the biomechanical effects of CXL remain measurable far beyond 2 years after surgery (48 ± 19 months on average). Larger scale studies are required to define when the biomechanical stabilization after CXL begins and how long it lasts postoperatively.
